# Prevalence of a large panel of systemic autoantibodies in the Bavarian adult population

**DOI:** 10.3389/fimmu.2024.1355905

**Published:** 2024-02-08

**Authors:** Florian Rohm, Elisabeth Kling, Reinhard Hoffmann, Christine Meisinger, Jakob Linseisen

**Affiliations:** ^1^ Epidemiology, Medical Faculty, University of Augsburg, Augsburg, Germany; ^2^ Institute for Laboratory Medicine and Microbiology, University Hospital Augsburg, Augsburg, Germany; ^3^ Institute for Medical Information Processing, Biometry, and Epidemiology (IBE), Ludwig-Maximilians-Universität München, München, Germany

**Keywords:** autoimmunity, autoantibody screening tests, systemic autoimmune antibodies, population-based study, BVS II, rheumatoid factor (RF), anti-ß2-glycoprotein 1 autoantibodies, anti-cardiolipin autoantibodies

## Abstract

**Objective:**

Autoimmune diseases commonly feature the presence of specific humoral autoantibodies. However, the prevalence of a large panel of systemic autoantibodies has never been assessed in the general population. We, therefore, described the prevalence of about 50 humoral systemic autoantibodies in a sample of the general Bavarian adult population.

**Methods:**

Non-fasting venous serum samples from 331 participants were analyzed for 7 autoantibody screening tests (nuclear, cytoplasmic, and mitotic ANA, ANCA, cANCA and pANCA, anti-ENA autoantibodies) and 44 different monospecific humoral non-organ specific/systemic autoantibodies using indirect immunofluorescence tests, ELISAs, and line blots. In order to assess associations between sex, age, BMI, education level, smoking status and the presence of systemic autoantibodies, logistic regression analyses were conducted.

**Results:**

At least one screening test was positive in 29.9% of the participants, and 42.3% of the participants were seropositive for at least one monospecific autoantibody. The most frequently found monospecific autoantibodies were rheumatoid factor (35.6%), ß2-glycoprotein 1 IgM (4.8%), and cardiolipin IgG (1.8%). Only few associations between sex, age, BMI, education, smoking status and autoantibody frequencies were observed.

**Conclusion:**

Systemic autoantibodies are common in the general Bavarian population, and largely independent of sex, age, BMI, education, or smoking status. The study results may give orientation to clinicians about the occurrence of autoantibodies in the population, not (yet) associated with clinical symptoms.

## 1 Introduction

Autoantibodies are antibodies directed against endogenous antigens. Non-pathogenic autoantibodies, so-called natural autoantibodies, are common in humans in serum from birth, i.e., prior to antigen exposure (1, 2). These natural autoantibodies are mostly oligo-specific IgM with generally low affinity, self-binding to different, often unrelated antigens. By binding to autoantigens in damaged, senescent, and apoptotic cells and thus facilitating their removal via phagocytosis, natural autoantibodies contribute to immune homeostasis and help prevent the development of self-immunity and atherosclerosis, and, via reacting to neoantigens, also of cancer (3). However, dysregulation or mutation of certain natural autoantibodies may be involved in the pathogenesis of autoimmune diseases (AIDs) (3, 4).

An overall increase in the incidence and prevalence of AIDs, particularly in Western civilization, has been observed in the past decades, becoming an important cause of morbidity and mortality (5). Besides the most commonly occurring autoimmune disorders autoimmune thyroiditis (AIT), celiac disease, rheumatoid arthritis (RA), systemic lupus erythematosus (SLE), and type 1 diabetes (T1D), the group of AIDs comprises far more than 80 different currently known pathologies (5). AIDs are commonly distinguished into organ-specific, like T1D, and systemic, like RA and SLE. All AIDs have in common that their precise etiology is unknown (5). The hypothesis that the combination of a genetic predisposition, modulated by epigenetics, as well as environmental factors, including nutrition, infections, xenobiotics, and pharmaceuticals underlie the triggering of AIDs, is generally acknowledged (5, 6).

Regardless of their genetic and environmental triggers, AIDs commonly feature the presence of specific humoral autoantibodies against defined antigens (7). However, many serum autoantibodies, e.g., antinuclear antibodies (ANA) and rheumatoid factor (RF) are also often observed in healthy individuals (8). Furthermore, in patients with autoimmune disorders, autoantibody plasma concentrations may vary over time, depending on the stage of the AID and its treatment (9–11). Elevated autoantibody levels may only be detectable transiently, like in celiac disease, where humoral autoantibodies can return to normal levels as long as the individual avoids the trigger of the disease (12).

To date, the prevalence of selected autoantibodies or small panels of systemic and/or organ-specific autoantibodies has been examined in the general population (13, 14), in disease-free populations (15), or in specific parts of the population, like in children (16), pregnant women (17), or the elderly (18). These studies observed that the majority of the general population appears not to have any detectable humoral autoantibodies, and that autoantibody prevalence increases with age. Like in AIDs, higher prevalence and overall higher levels of autoantibodies were found in women (19). However, to the best of our knowledge, the prevalence of a large panel of systemic autoantibodies has never been assessed in the general population. For this reason, we aimed to determine the prevalence of about 50 humoral systemic autoantibodies in a sample of the general Bavarian adult population. In addition, the association between sex, age, BMI, education level, and smoking status and the presence of systemic autoantibodies will be explored.

## 2 Methods

### 2.1 Study design and data collection

The second Bavarian Food Consumption Survey (BVS II) was designed as a representative cross-sectional study that aimed to describe the dietary and lifestyle habits of the German-speaking Bavarian population. The BVS II was conducted from 2002 to 2003, and 1050 subjects aged 13-80 years were recruited from private homes, with a participation rate of 71%. All participants gave their written informed consent. The BVS II was approved by the ethics committee of the Bavarian Medical Association (Bayerische Landesärztekammer) and conducted in accordance with the Declaration of Helsinki.

The study protocol included a computer-assisted personal interview (CAPI) assessing information on the subjects’ socio-economic status, lifestyle, and medical history, as well as three 24-h dietary recalls via computer-assisted telephone interviews (CATI). Further detailed information on the BVS II can be found elsewhere (20).

Adult participants who completed the CAPI and at least one CATI (n=879) were invited to a study center (i.e., the nearest health office) for blood sampling and anthropometric measurements, including body height, weight, and waist circumference. Blood samples and anthropometric measurements could be obtained from 65% of the invited participants (n=568). The participants’ blood samples did not all yield the same number of aliquots. For each type of blood sample, sets were compiled successively, resulting in the first sets being complete and later sets not containing aliquots from all participants. Due to financial constraints and the stipulation to use an unthawed set of samples, a later serum set was used for these analyses, which we do not expect to cause any bias.

Participants were categorized according to their BMI into groups of <18.5 kg/m^2^, 18.5-<25 kg/m^2^, 25-<30 kg/m^2^, and ≥30 kg/m^2^. The participants’ education levels were defined as “low” in case of primary and secondary general school and secondary school without a certificate; as “medium” in case of secondary school leaving certificate or comparable; and as “high” in case of higher education entrance qualification or university of applied sciences entrance qualification. Smoking status was categorized into “never smokers”, “former smokers”, and “current smokers”. With regard to physical activity, participants who self-reported regular sports activities during the last 12 months were considered as “active”, while participants with no regular sports activity were categorized as “inactive”. In addition, the participants’ physical activity was quantified by means of three 24-h recalls (21): Based on the type and intensity of the assessed activities, metabolic equivalents of task (METs) were determined and MET-hours per day were estimated on the basis of their duration. Participants were asked if they had ever been diagnosed with any of a set of conditions and diseases (hypertension, myocardial infarction, stroke, gastric and duodenal ulcer, inflammatory bowel disease, colorectal polyps, diabetes mellitus, hypercholesterolemia or hypertriglyceridemia, gout or hyperuricemia, osteoporosis, asthma, allergic rhinitis, allergic dermatitis, contact allergy, neurodermitis, psoriasis, food allergy, thyroid disease, depression, psychosis, addiction, malignant tumor), and if any medication had been prescribed for these conditions and diseases.

### 2.2 Laboratory methods

Non-fasting venous blood samples were collected, chilled at 4°C, centrifuged (within a maximum of 3 hours), aliquoted, and stored at -80°C until analysis. For the present study, serum samples from 331 BVS II participants were analyzed for 44 different humoral autoantibodies and 7 screening tests (**Table 1**).

**Table 1:** Classification of screening tests and monospecific autoantibodies by autoimmune disorders.

PANEL/AUTOIMMUNE DISORDER	AUTOANTIBODIES
Systemic rheumatic diseases - screening (Scr)	ANA (nuclear), ANA (mitotic), ANA (cytoplasmic), ENA, ANCA, cANCA, pANCA
Systemic sclerosis profile (SS)	CENP A, CENP B, fibrillarin, Ku, NOR90, PDGFR, PM-Scl75, PM-Scl100, Ro-52, RP11, RP155, Scl-70, Th/To
ANA profile (ANA)	AMA M2, CENP B, dsDNA, histones, Jo-1, Ku, Mi-2, nRNP/Sm, nucleosomes, PCNA, PM-Scl100, ribosomal P-proteins, Ro-52, Scl-70, Sm, SS-A, SS-B
Rheumatoid arthritis (RA)	CCP, RF
Vasculitis (V)	MPO, PR3
Autoimmune inflammatory myopathies (AIM)	EJ, Jo-1, Ku, MDA5, Mi-2 alpha, Mi-2 beta, NXP2, OJ, PL-7, PL-12, PM-Scl75, PM-Scl100, Ro-52, SAE1, SRP, TIF1g
Anti-phospholipid syndrome (APS)	cardiolipin (AMA M1) IgG, cardiolipin (AMA M1) IgM, ß2-glycoprotein 1 IgG, ß2-glycoprotein 1 IgM

All indirect immunofluorescence tests (IIFTs), ELISAs, and line blots were purchased from EUROIMMUN, Lübeck, Germany. All tests were performed fully automatically and evaluated on analysis devices from EUROIMMUN (Analyzer, Euroblotone, and Sprinter XL) following the manufacturer´s instruction. The IIFTs were evaluated using a fluorescence microscope. All assays were performed following the manufacturer’s instructions with sera at a 1:100 dilution. For evaluation, cut-off values were chosen as specified by the manufacturer (see **Supplementary Table 1**).

#### 2.2.1 IIFT

Screening of ANA and assessment of nuclear, mitotic, and cytoplasmic patterns according to International Consensus on ANA Patterns (ICAP) initiative (22) were performed by IIFTs using human epithelial cells (HEp-2) and liver tissue provided by EUROIMMUN. Positive cut-off values were >1:100, while titers of 1:100 were considered borderline.

Screening of anti-neutrophil cytoplasm antibodies (ANCA) associated with different types of autoimmune vasculitis and subsequent differentiation of positive ANCA tests into cytoplasmic ANCA (cANCA), often associated with granulomatosis with polyangiitis and usually directed against PR3, and perinuclear ANCA (pANCA), which are associated with various diseases, including microscopic polyangiitis and eosinophilic granulomatosis with polyangiitis, and often constitute anti-MPO antibodies, were performed by IIFT using ethanol-fixed human granulocytes provided by EUROIMMUN. Positive cut-off values were >1:10, while titers of 1:10 were considered borderline.

#### 2.2.2 ELISA

Serum levels of the following autoantibodies, and antibodies against the following antigens, respectively, were assessed using commercial ELISAs from EUROIMMUN:

Extractable nuclear antigens (ENA), double-stranded DNA (dsDNA), myeloperoxidase (MPO), proteinase 3 (PR3), IgM rheumatoid factor (RF), cyclic citrullinated peptides (CCP), cardiolipin IgG and IgM, and ß2-glycoprotein 1 IgG and IgM.

Anti-ENA antibody screening was performed based on an antigen mixture of nRNP/Sm, Sm, SS-A, SS-B, Scl-70, and Jo-1.

Cut-off values were 100 U/ml for dsDNA, 20 U/ml for ENA, MPO, PR3, ß2-glycoprotein 1 IgG, and ß2-glycoprotein 1 Ig M, 14 U/ml for RF, 12 U/ml for cardiolipin IgG and IgM, and 5 U/ml for CCP.

#### 2.2.3 Line blot

The following ANAs, autoantibodies that produce a cytoplasmic pattern on HEp-2 cells, as well as autoantibodies associated with autoimmune inflammatory myopathies and systemic sclerosis were tested by line blots from EUROIMMUN:

AMA M2, CENP A, CENP B, EJ, fibrillarin, histones, Jo-1, Ku, MDA5, Mi-2, Mi-2 alpha, Mi-2 beta, NOR90, nRNP/Sm, nucleosomes, NXP2, OJ, PCNA, PDGFR, PL-12, PL-7, PM-Scl100, PM-Scl75, ribosomal P-proteins, Ro-52, RP11, RP155, SAE1, Scl-70, Sm, SRP, SS-A, SS-B, Th/To, and TIF1g.

### 2.3 Statistical analysis

The distribution of continuous data was assessed with the Shapiro-Wilk test. Welsh’s *t*-test was used to compare the means of normally distributed phenotypic characteristics between men and women, while the Mann-Whitney *U* test was used otherwise. Fisher’s exact test was applied to compare categorical characteristics. Logistic regression analyses were performed to assess associations between the presence of autoantibodies (outcome) and phenotypic traits (exposure). For this purpose, the positivity of single autoantibody tests as well as autoantibody group positivity were considered as binomial variables. Linear regression analyses were performed to assess associations between the number of positive autoantibody screening tests and the number of positive monospecific autoantibody tests per participant, respectively, and phenotypic traits (exposure). In all regression models, sex, education level, and smoking status were considered categorical variables, while the participants’ age and BMI were considered continuous variables and were tested for linearity. For all tests, *p* values <0.05 were considered significant. All statistical analyses were performed with IBM SPSS Statistics 28.0.

## 3 Results

### 3.1 Characteristics of the study sample

The characteristics of the total sample and by sex (331 participants; men: n=129, 39.0%; women: n=202, 61.0%) are shown in **Table 2**. The participants’ mean age was 47.9 (± 15.5) years, with male participants on average being older than female participants (50.4 ± 16.1 vs. 46.3 ± 15.0, *p*=0.017). BMI, physical activity (MET-hours per day), the prevalence of 1 or more pre-existing diseases and the intake of corresponding medications did not differ significantly between men and women. Mean waist circumference (100.4 ± 11.8 vs. 89.1 ± 13.9 cm, *p*<0.001) and median alcohol consumption were greater in men, while median physical activity in MET-hours per day was greater in women (37.8 (35.3 - 41.9) vs. 36.3 (33.7 - 42.4), *p*=0.043). Women were more likely to be never smokers, while men were more likely to be current smokers. Medium-level education was more prevalent in women, while high-level education was more prevalent in men.

**Table 2:** Characteristics of the study population.

Characteristics	total (n=331)	male (n=129)	female (n=202)	*p* value (male vs. female)
Age at recruitment (years)	47.9 ± 15.5	50.4 ± 16.1	46.3 ± 15.0	0.017
Age groups				0.042
18-<30 years	38 (11.5%)	14 (10.9%)	24 (11.9%)	
30-<40 years	79 (23.9%)	25 (19.4%)	54 (26.7%)	
40-<50 years	68 (20.5%)	20 (15.5%)	48 (23.8%)	
50-<65 years	92 (27.8%)	42 (32.6%)	50 (24.8%)	
>=65 years	54 (16.3%)	28 (21.7%)	26 (12.9%)	
BMI (kg/m^2^)	26.4 ± 4.9	26.9 ± 4.2	26.1 ± 5.2	0.011
BMI groups				0.429
BMI <18.5 kg/m²	5 (1.5%)	1 (0.8%)	4 (2.0%)	
BMI 18.5-<25 kg/m²	146 (44.1%)	51 (39.5%)	95 (47.0%)	
BMI 25-<30 kg/m²	117 (35.3%)	51 (39.5%)	66 (32.7%)	
BMI >=30 kg/m²	63 (19.0%)	26 (20.2%)	37 (18.3%)	
Waist circumference (cm)	93.6 ± 14.2	100.4 ± 11.8	89.1 ± 13.9	<0.001
Education				0.001
Low	133 (40.2%)	58 (45.0%)	75 (37.1%)	
Medium	111 (33.5%)	27 (20.9%)	84 (41.6%)	
High	71 (21.5%)	35 (27.1%)	36 (17.8%)	
Missing	16 (4.8%)	9 (7.0%)	7 (3.5%)	
Smoking status				0.003
Never smoker	182 (55.0%)	56 (43.4%)	126 (62.4%)	
Former smoker	66 (20.0%)	31 (24.0%)	35 (17.3%)	
Current smoker	83 (25.1%)	42 (32.6%)	41 (20.3%)	
Alcohol intake (g/day)*	7.0 (0.1-20.1)	15.8 (1.3-33.2)	3.9 (0.0-12.7)	<0.001
Missing	4 (1.2%)	3 (2.3%)	1 (0.5%)	
Physical activity				0.347
Yes	149 (45.0%)	63 (48.8%)	86 (42.6%)	
No	153 (46.2%)	56 (43.4%)	97 (48.0%)	
Missing	29 (8.8%)	10 (7.8%)	19 (9.4%)	
Physical activity in MET-hours per day*	37.0 (34.7-41.9)	36.3 (33.7-42.4)	37.8 (35.3-41.9)	0.043
Pre-existing illness (1 or more)	206 (62.2)	76 (58.9%)	130 (64.4%)	0.487
Inflammatory bowel disease	5 (1.5%)	2 (1.6%)	3 (1.5%)	
Psoriasis	14 (4.2%)	5 (3.9%)	9 (4.5%)	
Medication (1 or more)	167 (50.5)	62 (48.1)	105 (52%)	0.523

Values are reported as arithmetic mean ± standard deviation (SD), * median (25^th^ – 75^th^ percentile), or n (%); p values were derived from Student’s t-tests (means), Mann-Whitney U tests (medians), and Fisher’s exact tests, respectively. Abbreviations: BMI, body mass index; MET, metabolic equivalent of task.

### 3.2 Prevalence of positive autoantibody screening tests

At least 1 autoantibody screening test (for systemic rheumatic diseases) was positive in 29.9% of the participants (**Table 3**). The prevalence of positive nuclear ANA was 12.4%, of mitotic ANA 1.5%, and of cytoplasmic ANA 3.3%. Only a single participant tested positive for more than 1 ANA pattern, nuclear and mitotic. The majority of nuclear patterns was AC-4/5 (n=26), while 7 participants featured AC-8/910 and 2 participants each AC-7 and AC-11/12 (**Table 4**). The observed cytoplasmic patterns consisted of AC-15/16/17 (n=2), AC-19/20 (n=3), AC-21 (n=5), and AC-22 (n=1). In terms of mitotic patters, 3 participants featured AC-24 and 2 AC-25.

**Table 3:** Results of the autoantibody screening tests.

		total (n=331)		Sex			
				male		female	
				(n=129)		(n=202)	
		n	%	n	%	n	%
Systemic rheumatic diseases - screening	negative	232	70.1	99	76.7	133	65.8
	positive	99	29.9	30	23.3	69	34.2
ANA, nuclear	negative	220	66.5	91	70.5	129	63.9
	borderline	70	21.1	26	20.2	44	21.8
	positive	41	12.4	12	9.3	29	14.4
ANA, cytoplasmic	negative	304	91.8	123	95.3	181	89.6
	borderline	15	4.5	4	3.1	11	5.4
	positive	11	3.3	2	1.6	9	4.5
ANA, mitotic	negative	326	98.5	128	99.2	198	98.0
	borderline	0	0	0	0	0	0
	positive	5	1.5	1	0.8	4	2.0
ANCA	negative	280	84.6	113	87.6	167	82.7
	borderline	0	0	0	0	0	0
	positive	51	15.4	16	12.4	35	17.3
cANCA	missing	280	84.6	113	87.6	167	82.7
	negative	44	13.3	14	10.9	30	14.9
	borderline	6	1.8	2	1.6	4	2.0
	positive	1	0.3	0	0	1	0.5
pANCA	missing	280	84.6	113	87.6	167	82.7
	negative	7	2.1	2	1.6	5	2.5
	borderline	26	7.9	10	7.8	16	7.9
	positive	18	5.4	4	3.1	14	6.9
anti-ENA antibodies	negative	326	98.5	128	99.2	198	98.0
	borderline	0	0	0	0	0	0
	positive	5	1.5	1	0.8	4	2.0

**Table 4:** ANA patterns in borderline and positive ANA screening tests according to the ICAP nomenclature.

ANA screening IIFT		
	borderline	positive (titer range)
**Nuclear pattern, total**	**70**	**41**
AC-1	0	4
AC-4/5	52	26 (1:200-1:400)
AC-7	0	2 (1:200)
AC-8/9/10	18	7 (1:200)
AC-11/12	0	2 (1:400)
**Cytoplasmic pattern, total**	**15**	**11**
AC-15/16/17	6	2 (1:200-1:400)
AC-18	1	0
AC-19/20	4	3 (1:200-1:400)
AC-21	4	5 (1:200)
AC-22	0	1 (1:200)
**Mitotic pattern, total**	**0**	**5**
AC-24	0	3 (1:200-1:400)
AC-25	0	2 (1:200)

AC-1, nuclear homogenous; AC-4, nuclear fine speckled; AC-5, nuclear large/coarse speckled; AC-7, few nuclear dots; AC-8, homogenous nucleolar; AC-9, clumpy nucleolar; AC-10, punctate nucleolar; AC-11, smooth nuclear envelope; AC-12, punctate nuclear envelope; AC-15, cytoplasmic linear; AC-16, cytoplasmic filamentous; AC-17, cytoplasmic segmetal; AC-18, cystoplasmic discrete dots; AC-19, cytoplasmic dense fine speckled; AC-20, cytoplasmic fine speckled; AC-21, cytoplasmic reticular; AC-22, cytoplasmic polar; AC-24, centrosome; AC-25, spindle fibers.

Bold values are the total values for each pattern.

Anti-ENA antibody screening was positive in 1.5% of the participants (n=5). Mitotic and cytoplasmic ANA were not detected in any of these 5 participants, while 3 tested positive and 2 tested borderline positive for nuclear ANA. All 5 participants tested positive for exactly 1 monospecific autoantibody of the ENA panel. In neither of the 5 participants, autoantibodies against nRNP/Sm, SM, nor Jo-1 could be detected. 3 of the participants were positive for anti-SS-A, 2 for anti-Scl-70, and 1 participant was borderline for anti-SSB antibodies. Of the 326 participants with negative anti-ENA antibody screening tests, anti-nRNP/Sm antibodies were detected in 1 participant, and anti-SS-A antibodies in 1 other participant.

ANCA screening revealed 15.4% (n=51) ANCA positive participants, of whom 1 tested positive for cANCA, 6 were borderline for cANCA, 18 were pANCA positive, and 26 were pANCA borderline; none of these participants tested positive for both cANCA and pANCA (**Table 3**). Neither of the 7 cANCA positive or borderline participants tested positive for monospecific anti-PR3 antibodies, and neither of the 44 pANCA positive or borderline participants tested positive for monospecific anti-MPO antibodies either (of the 280 ANCA negative participants, 1 was anti-MPO positive and 1 was both anti-MPO and anti-PR3 positive).

### 3.3 Prevalence of systemic monospecific autoantibodies, by groups

35.6% of the participants tested positive for at least 1 autoantibody associated with rheumatoid arthritis, 7.3% for 1 or more autoantibodies associated with anti-phospholipid syndrome, 3.6% for 1 or more autoantibodies from the ANA profile, 3.0% for at least 1 autoantibody associated with autoimmune inflammatory myopathies (AIM), and 2.1% for 1 or more autoantibodies associated with systemic sclerosis (**Table 5**). Only 2 participants showed vasculitis-associated autoantibodies.

**Table 5:** Prevalence of one or more positive monospecific autoantibodies, by autoantibody groups associated with autoimmune disorders.

Autoimmune disorder	total (n=331)		male (n=129)		female (n=202)	
	n	%	n	%	n	%
Systemic sclerosis profile	7	2.1	2	1.6	5	2.5
ANA profile	12	3.6	3	2.3	9	4.5
Rheumatoid arthritis	118	35.6	44	34.1	74	36.6
Vasculitis	2	0.6	0	0.0	2	1.0
Autoimmune inflammatory myopathies	10	3.0	5	3.9	5	2.5
Anti-phospholipid syndrome	24	7.3	5	3.9	19	9.4

### 3.4 Number of systemic monospecific autoantibodies, by participant

In 42.3% of the participants (n=140), at least 1 monospecific autoantibody could be detected. 32.6% of all participants featured 1 autoantibody, 8.5% 2 autoantibodies, 0.9% 3 autoantibodies, and 5 autoantibodies were observed in 1 participant (0.3%) (**Table 6**).

**Table 6:** Frequency of systemic monospecific autoimmune disease-related autoantibodies per participant.

Number of detected autoantibodies	total (n=331)		Sex			
			male (n=129)		female (n=202)	
	n	%	n	%	n	%
0	191	57.7	78	60.5	113	55.9
1	108	32.6	42	32.6	66	32.7
2	28	8.5	8	6.2	20	9.9
3	3	0.9	1	0.8	2	1.0
5	1	0.3	0	0.0	1	0.5

Amongst all tested autoantibodies, 25 were not positive in any participant (see **Supplementary Table 2**). Otherwise, the prevalence of single autoantibodies ranged between 0.3% (n=1, for e.g., anti-CENP A and anti-PM-Scl75) and 35.6% (rheumatoid factor). The 5 most frequently found types of monospecific autoantibodies were RF (35.6%, n=118), ß2-glycoprotein 1 IgM (4.8%, n=16), cardiolipin IgG (1.8%, n=6), cardiolipin IgM (1.5%, n=5), and anti-dsDNA (1.5%, n=5).

### 3.5 Results of the regression analyses

Regression analysis did not reveal any significant association between sex, age, BMI, and education and positive autoantibody screening prevalence. However, current smokers tested significantly less often positive for autoantibody screening tests than never smokers (**Tables 7**, **8**). Sex, age, BMI, education, and smoking status were not associated with the prevalence of any single screening test positivity (see **Supplementary Table 3**).

**Table 7:** Results of the linear regression analysis: associations between participants’ characteristics and the number of positive screening tests and the number of positive monospecific autoantibody tests.

Outcome	Characteristics	β	95% CI		*p* value
			Lower limit	Upper limit	
Number of positive autoantibody screening tests	Female sex	0.105	-0.015	0.263	0.079
	Age	0.012	-0.004	0.005	0.838
	BMI	0.048	-0.008	0.020	0.423
	Medium-level education	-0.011	-0.162	0.136	0.863
	High-level education	0.005	-0.162	0.177	0.933
	Former smoker	-0.045	-0.231	0.103	0.453
	Current smoker	-0.140	-0.347	-0.025	0.024
Number of positive monospecific autoantibody tests^1^	Female sex	0.091	-0.032	0.244	0.131
	Age	0.032	-0.003	0.006	0.601
	BMI	-0.103	-0.026	0.002	0.087
	Medium-level education	-0.116	-0.285	0.011	0.070
	High-level education	-0.081	-0.278	0.059	0.202
	Former smoker	-0.016	-0.189	0.143	0.785
	Current smoker	0.022	-0.132	0.189	0.725

Reference groups were males, low-level education, and never smokers; ^1^ square root of outcome data.

**Table 8:** Results of the logistic regression analysis: associations between participants’ characteristics and autoantibody screening, anti-phospholipid syndrome-associated autoantibody, and ß2-glycoprotein 1 IgM positivity.

Outcome	Characteristics	Odds ratio	95% CI		*p* value
			Lower limit	Upper limit	
Autoantibody screening positivity	Female sex	1.542	0.888	2.676	0.124
	Age	1.002	0.984	1.020	0.830
	BMI	1.019	0.966	1.074	0.490
	Medium-level education	0.807	0.451	1.444	0.471
	High-level education	0.951	0.492	1.837	0.880
	Former smoker	0.770	0.406	1.460	0.423
	Current smoker	0.449	0.226	0.890	0.022
Anti-phospholipid syndrome	Age	1.021	0.990	1.053	0.184
	Female sex	4.194	1.304	13.488	0.016
	BMI	0.924	0.829	1.031	0.157
	Medium-level education	0.289	0.087	0.965	0.044
	High-level education	1.124	0.393	3.218	0.828
	Former smoker	0.561	0.119	2.654	0.466
	Current smoker	1.729	0.611	4.890	0.302
ß2-glycoprotein 1 IgM	Female sex	1.042	1.003	1.082	0.035
	Age	2.757	0.776	9.796	0.117
	BMI	0.963	0.852	1.088	0.544
	Medium-level education	0.622	0.168	2.303	0.477
	High-level education	1.262	0.333	4.782	0.732
	Former smoker	1.091	0.208	5.711	0.918
	Current smoker	3.478	1.006	12.022	0.049

Reference groups were males, low-level education, and never smokers.

(Results for all autoantibodies are given in the **Supplementary Tables 3**, **4**).

The number of positive autoantibody tests was not associated with sex, age, BMI, education, and smoking status (**Table 7**).

The prevalence of anti-phospholipid syndrome autoantibodies was increased in women compared to men and reduced in medium-level compared to low-level education (**Table 8**). We did not observe any association between sex, age, BMI, education, and smoking status and autoantibody prevalence in the autoantibody groups associated with AIM, rheumatoid arthritis, systemic sclerosis, or vasculitis, nor the ANA profile (see **Supplementary Table 3** with the results of the full analysis).

The prevalence of ß2-glycoprotein 1 IgM was significantly associated with age and smoking status (**Table 8**). The prevalence of the other most common monospecific autoantibodies, RF, cardiolipin IgG and IgM, and anti-dsDNA was not associated with sex, age, BMI, education and smoking status (see **Supplementary Table 4** with the results of the full analysis).

## 4 Discussion

The present study revealed that 29.9% of adults from the general Bavarian population tested positive for at least 1 screening test for systemic rheumatic diseases, while 42.3% of the study participants tested positive for at least 1 of the evaluated monospecific autoantibodies of our panel. Contrary to previous studies assessing the prevalence of specific autoantibodies, e.g., [cf (13, 15, 18, 19)], we could not observe an overall higher systemic autoantibody prevalence in women (versus men) nor an increase with higher age. Bar few exceptions, also BMI, the level of education, and smoking status were not related to the prevalence of specific autoantibodies or groups of autoantibodies.

Literature provides a decent amount of data regarding the prevalence of many autoantibodies in specific parts of the population, like in children, e.g., [cf (16)], pregnant women, e.g., [cf (17)], or the elderly, e.g., [cf (18)]. So far, only few studies determined the population prevalence of a small number of autoantibodies. Haller-Kikkatalo and coworkers examined the prevalence of autoantibodies in Estonian adults (n=994) without AIDs (15). Compared to our study, the autoantibody panel only comprised the following 5 autoantibody tests: anti-thyroid peroxidase (TPO), anti-issue transglutaminase (tTG), anti-CCP, anti-glutamic acid decarboxylase (GADA), and a connective tissue disease (CTD) screening test; borderline and positive CTD tests were further specified into anti-dsDNA, anti-SS-A, anti-SS-B, anti-CENP, anti-Jo-1, anti-Scl-70, anti-Sm, and anti-U1RNP. The highest monospecific autoantibody prevalences were 8.8% for anti-GADA, 7.2% for anti-TPO, and 7.2% for anti-dsDNA, while all other prevalences were well below 1%. The observed overall autoantibody prevalence was 23.6% and, unlike in our study, female sex and higher age were associated with increased frequencies.

Another study assessed autoantibody prevalence in the US general population, based on data from the U.S. National Health and Nutrition Examination Surveys (NHANES; numbers of examined participants ranged between n=3,863 and 25,871) (13). Here, the autoantibody panel comprised the organ-specific thyroid autoantibodies anti-thyroglobulin (anti-TG) and anti-TPO, the celiac disease-associated anti-tTG and, in case of positive anti-tTG, anti-endomysial autoantibodies (anti-EMA), as well as autoantibodies to the 65-kDa isoform of glutamic acid decarboxylase (anti-GAD65) associated with diabetes. In addition, the prevalence of systemic autoantibodies was assessed for RF, ANA, and, in case of positive ANA, anti-ENA autoantibodies, a subset of ANA used to diagnose, differentiate, and monitor various autoimmune disorders and associated with different CTDs, including SLE, Sjögren’s syndrome, and different forms of systemic sclerosis (23). Similar to our results, the study overall revealed a substantially high autoantibody prevalence in the US population, however, the prevalence was higher in women (39% vs. 22% in men) and older individuals, an observation that we could not confirm in our regression analyses.

The prevalence of RF, an antibody directed against the Fc portion of IgG and associated with rheumatoid arthritis, in the general population as reported in the literature varies: For instance, in Northern Italy, an RF prevalence of 8.1% has been described (24), while in Denmark, 17% of the general population tested positive for RF (25). RF is not limited to rheumatoid arthritis though, but can also be found in other AIDs like Sjögren’s syndrome, infectious diseases like hepatitis and tuberculosis, as well as in healthy individuals (26). Regarding the prevalence of anti-CCP autoantibodies, another autoantibody type associated with RA, similarly differing results can be found. In Northern-Italy, a prevalence of 4.8% has been described (24), while anti-CCP seropositivity was reported for 1.0% of the general population in the Netherlands (27) and in 0% of healthy controls in Thailand (28). Overall, the prevalence of RF we observed in the general population was distinctly higher compared to literature, while the prevalence of CCP in our study was similar.

Low population frequencies of autoantibodies associated with anti-phospholipid syndrome (APS) have been reported in literature: For cardiolipin IgM, prevalence generally ranges between 1 and 5%, and for cardiolipin IgG between 1 and 4.2% (29). In the Australian population, ß2-glycoprotein 1 IgM were found in 9.6%, and the prevalence of cardiolipin IgG was 3.5% (30), while a prevalence of autoantibodies against ß2-glycoprotein 1 (combined IgA, IgM, and IgG) of 1.3% in healthy controls was reported for Israel (31). Our observations regarding a low prevalence of APS-associated autoantibodies corresponded to these findings.

Autoantibodies against fibrillarin, Th/To, RP155, NOR90, and PM-Scl75 are types of ANA that are associated with systemic sclerosis and, due to their specificity for this autoimmune disorder, employed in the prediction of the disease’s clinical manifestations (32). ANA, which target, for instance, antigens like nucleic acids and nuclear and ribonuclear proteins, are important serological markers for CTDs and are generally not uncommon in the general population with a prevalence of up to 30% (33). However, the prevalence of the monospecific autoantibodies in the general population had not been systematically assessed previously. A Chinese study reported frequencies of 0% for anti-Th/To, anti-fibrillarin, and anti-RP155, and 3.3% for anti-NOR90 and anti-PM-Scl75 in healthy controls (n=30) (34), while in a Malaysian and in a Turkish study, anti-dsDNA autoantibodies could not be detected in healthy individuals (35, 36). The frequency of seropositivity of these ANA we observed in our sample was similarly low.

Certain types of autoantibodies are used as clinical markers for different types of AIM. These autoantibodies comprise myositis-specific autoantibodies (MSAs) present in up to 70% of AIM patients (37, 38), including, e.g., anti-Mi-2, anti-SRP, and anti-TIF1g, as well as myositis-associated autoantibodies (MAAs), which may also be found in other AIDs like SLE or systemic sclerosis, including anti-PM-Scl. Like for autoantibodies associated with systemic sclerosis, information regarding the prevalence of MSAs and MAAs in the general population is scarce. In a recent study assessing MSA and MAA prevalence in COVID-19 patients, an overall MSA and MAA prevalence of 2.4% was determined in healthy controls (n=41), yet neither anti-PM-Scl75, nor anti-Mi-2 beta, anti-SRP, and anti-TIF1g were positive in any controls (39). In our study population, the prevalence of these AIM-associated autoantibodies was comparably low, ranging between 0% (e.g., anti-SRP) and 1.2% (anti-Mi-2 beta).

Current smokers testing positive significantly less often for autoantibody screening tests than never smokers came as a surprise as many studies have demonstrated an increased prevalence of AIDs in smokers compared to non-smokers, e.g., for the systemic AIDs RA (40) and SLE (41). However, negative associations between smoking and autoantibody prevalence have been described as well, e.g., for SLE autoantibodies (42, 43) and for thyroid autoantibodies (44). Also, similarly to our findings, ANA prevalence has been shown to be inversely associated with the frequency of smoking (45, 46), while other studies did not observe any association between smoking and ANA prevalence (47). Overall, data regarding the interaction of smoking and autoantibody prevalence appear to be conflicting. The reasons for the prevalence of certain autoantibodies being reduced by smoking in some studies is not known.

Overall, we observed a similar prevalence of monospecific autoantibodies as reported in the literature, with the exception of RF. The reasons for this discrepancy are unknown – they could include most likely different test performances (sensitivity, specificity, etc.), differences in the age structure of the general population, or the regional differences in autoantibody prevalence mentioned above.

Although a growing number of autoantibodies that are specific for or associated with certain AIDs are being discovered, it is not yet possible to infer an individual’s probability of developing an autoimmune disease from the presence of the corresponding associated autoantibodies, as autoantibodies may also be present in healthy subjects, or they may not be detectable in subjects suffering from the corresponding AID (12), as mentioned above. Thus, the prevalence of autoantibody groups that we presented can be assumed to be distinctly higher than the prevalence of the corresponding AID: For instance, the prevalence of RA in Germany is 0.8%, with women being affected 3 times more often than men (48), while we determined RA-associated autoantibodies in 35.6% of the sample, without pronounced differences between men and women (34.1% and 36.6%, respectively). Cures for autoimmune disorders are currently unavailable, making primary and secondary prevention of AIDs all the more important. The screening of autoantibodies as important risk factors for the development of AIDs in healthy individuals, i.e., before clinical symptoms manifest, may be one way to achieve that. However, this still requires a better understanding of which autoantibodies can serve as prognostic markers for the development of an AID, i.e., which seropositive healthy persons with preclinical autoantibodies will develop clinical symptoms and which individuals will remain disease-free, which can only be answered by long-term follow-up studies.

### 4.1 Strengths and weaknesses

The present data for the first time describes the frequencies of a large panel of systemic autoantibodies in the general population, covering antibodies specific for or associated with the most common systemic rheumatic diseases, including rheumatoid arthritis, systemic sclerosis, and autoimmune inflammatory myopathies. For many of our panel autoantibodies, no information regarding their prevalence in the general population was previously available at all. As such, the prevalence of autoantibody positivity as determined in the adult Bavarian population may serve as a reference for other studies, including healthy subjects or AID patients in the future.

Due to the cross-sectional design of our study, our findings cannot be used to infer causality, as a temporal sequence cannot be established. In addition, the specific composition of the Bavarian population in terms of age and race does not enable a transfer of the results to other ethnicities and age groups, especially since there is no information available on the exact ethnic composition of the study participants. Another weakness of our study is the lack of information about the presence of AIDs, immunodeficiency, and immunosuppressive medication in study participants, which makes it impossible to split autoantibody prevalence data into healthy subjects and AID patients. In addition, analysis results may vary depending on the manufacturer’s test design and test performance, which could explain the different frequencies observed for certain autoantibodies in different studies. Notably, all test kits used in our analyses were purchased from the same provider, which may likely contribute to the higher prevalence of RF we observed compared to similar studies. Also, the autoantibody levels were not taken into account in this epidemiological evaluation. However, the autoantibody levels play an important role in the clinical evaluation (see **Supplementary Table 5** with the IIFT and ELISA ranges and medians).

### 4.2 Conclusion

Systemic autoantibodies are common in the general Bavarian population: A surprisingly high proportion of 42.3% featured one or more autoantibodies, with one individual testing positive for 5 different autoantibodies. One panel autoantibody, RF, associated with rheumatoid arthritis, was observed in more than a third of the study population. Considerable autoantibody frequencies were observed in both sexes as well as in all age groups, but they were, except for very few exceptions, not associated with sex, age, BMI, education, and smoking status.

## Data availability statement

The raw data supporting the conclusions of this article will be made available by the authors, without undue reservation.

## Ethics statement

The studies involving humans were approved by Ethics committee of the Bavarian Medical Association (Bayerische Landesärztekammer). The studies were conducted in accordance with the local legislation and institutional requirements. The participants provided their written informed consent to participate in this study.

## Author contributions

FR: Conceptualization, Formal analysis, Writing – original draft, Writing – review & editing. EK: Writing – review & editing. RH: Writing – review & editing. CM: Conceptualization, Resources, Writing – review & editing. JL: Conceptualization, Funding acquisition, Resources, Writing – review & editing.
